# Biogenesis of the oxidative phosphorylation machinery in plants. From gene expression to complex assembly

**DOI:** 10.3389/fpls.2014.00225

**Published:** 2014-05-23

**Authors:** Daniel H. Gonzalez, Philippe Giegé

**Affiliations:** ^1^Instituto de Agrobiotecnología del Litoral (CONICET-UNL), Universidad Nacional del LitoralSanta Fe, Argentina; ^2^Centre National de la Recherche Scientifique, Institut de Biologie Moléculaire des Plantes du CNRS (IBMP-CNRS), Université de StrasbourgStrasbourg, France

**Keywords:** cofactor assembly, coordinated expression, mitochondrion, mitoribosome, protein import translocase, respirasome, respiratory pathway, RNA editing

Mitochondrial biogenesis is an extremely complex process. A hint of this complexity is clearly indicated by the many steps and factors required to assemble the respiratory complexes involved in oxidative phosphorylation. These steps include the expression of genes present in both the nucleus and the organelle, intricate post-transcriptional RNA processing events, the coordinated synthesis, transport and assembly of the different subunits, the synthesis and assembly of co-factors and, finally, the formation of supercomplexes or respirasomes. Plants have evolved specific mechanisms for the biogenesis of respiratory complexes. For example, expression of the mitochondrial genome in plants has special features, not present in other groups of eukaryotes. Moreover, plant mitochondrial biogenesis and function should be considered in the context of the presence of the chloroplast, a second organelle involved in energetic and redox metabolism. This particularity implies the necessity to discriminate between proteins destined to each organelle and requires the establishment of functional interconnections between photosynthesis and respiration. In recent years, our knowledge of the mechanisms involved in these different processes in plants has considerably increased. As a result, the many events and factors necessary for the correct expression of proteins encoded in the mitochondrial genome, the *cis* acting elements and factors responsible for the expression of nuclear genes encoding respiratory chain components, the signals and mechanisms involved in the import of proteins synthesized in the cytosol and the many factors required for the synthesis and assembly of the different redox co-factors (heme groups, iron-sulfur clusters, copper centers) are beginning to be recognized at the molecular level (Carrie et al., 2013; Duncan et al., 2013; Garcia et al., 2014; Hammani and Giegé, 2014). However, detailed knowledge of these processes is still not complete and, especially, little is known about how these processes are interconnected. Regarding gene expression, key questions remain, e.g., the nature of the enzyme performing RNA editing remains elusive and the *cis* elements recruiting mitoribosomes are unknown. Other central questions regarding the post-translational fate of proteins are also unanswered, e.g., how the proteins, once synthesized in the mitochondrial matrix, are inserted into the membrane and assembled with other components, including those imported from the cytosol, how the expression of both genomes is coordinated and responds to changes in mitochondrial function, cellular requirements or environmental cues, or which factors and conditions influence the assembly of complexes and supercomplexes are still open and will receive much attention in the near future.

This Research Topic is aimed at establishing a collection of articles that focus on the different processes involved in the biogenesis of respiratory complexes in plants as a means to highlight recent advances. In this way, it intends to help to construct a picture of the whole process and, not less important, to expose the existing gaps that need to be addressed to fully understand how plant cells build and modulate the complex structures involved in respiration.

In the first article of this Topic, Schertl and Braun (2014) illustrate the complexity of plant mitochondrial electron pathways involved in respiration and describe the many dehydrogenases present in plant mitochondria and the entry points of electrons to the respiratory chain. Processes involved in the expression of the mitochondrial genome, like the role of maturases encoded in the organelle or the nucleus in the splicing of group II introns, RNA editing by PPR proteins and the translation of mitochondrial RNAs are discussed in the articles by Brown et al. (2014), Brehme et al. (2014) and Kazama et al. (2014), respectively. Welchen et al. (2014) present a survey of the current knowledge about the coordination of the expression of nuclear genes encoding mitochondrial components and how the expression is modulated by signals that arise in several compartments, including the organelle itself. In relation to this, Janska and Kwasniak (2014) discuss recent findings indicating the selective translation of sets of mitochondrial transcripts by mitoribosomes and the possible regulatory role of this phenomenon in the synthesis of mitochondrial proteins and the assembly of respiratory complexes, and Murcha et al. (2014) present new findings that mitochondrial Tim21-like proteins interact with both the protein import translocase of the inner membrane and respiratory chain complexes, reinforcing the idea that the functioning of the respiratory chain and the import and assembly of the corresponding subunits are tightly interconnected processes. In other review articles, Rigas et al. (2014) discuss the role of Lon proteases in mitochondrial biogenesis and analyze the evolutionary history of the Lon family and the processes that originated the current diversity of this family that includes a couple of dual-targeted (i.e., chloroplastic and mitochondrial) proteins, while Gehl and Sweetlove (2014) discuss on the role of Band-7 family proteins in mitochondrial biogenesis and the supramolecular organization and functioning of the respiratory chain through the possible formation of lipid microdomains. Finally, Steinebrunner et al. (2014) present results about the divergent role of plant Sco proteins, involved in metal cofactor insertion into respiratory Complex IV in other organisms, in mitochondrial biogenesis and stress responses.

In summary, the collection of articles of this Research Topic highlights the complexity of the mechanisms involved in the biogenesis of the plant mitochondrial respiratory chain and the unique features of this process in plants. We thank all authors for their contribution to the assembly of this issue and hope that it will be useful for a broad readership of students and researchers interested in both plant sciences and organelle biology.

## Conflict of interest statement

The authors declare that the research was conducted in the absence of any commercial or financial relationships that could be construed as a potential conflict of interest.
